# The L-A dsRNA Virus and Its Satellites: Totiviruses and Killers in *Saccharomyces cerevisiae*

**DOI:** 10.3390/v18080920

**Published:** 2026-08-21

**Authors:** Reed B. Wickner, Herman K. Edskes

**Affiliations:** Laboratory of Biochemistry and Genetics, National Institute of Diabetes and Digestive and Kidney Diseases, National Institutes of Health, Bethesda, MD 20892, USA; hermane@niddk.nih.gov

**Keywords:** dsRNA virus, Totivirus, polyA, Ski, anti-virus, killer, translation control, Nuc1

## Abstract

A secreted protein toxin encoded by a satellite dsRNA-enabled dissection of the genetic control of replication and expression of a single-segment dsRNA virus, the first Totivirus, L-A. Among the then-novel findings were i. “head-full replication”, ii. a supposedly forbidden “T = 2” capsid symmetry based on an asymmetric dimer, iii. a host N-acetyltransferase whose modification of the coat protein is necessary for packaging, iv. Kex1 and Kex2 pro-toxin peptidases leading to discovery of the pre-pro-insulin processing enzymes, and v. specific viral (+) strand sites/structures needed for RNA packaging and (-) strand synthesis. L-A viral (+) strands made in the particle are extruded to the cytoplasm. Those destined for translation are 5′ 7meGMP-capped by a coat protein activity that steals the cap from cellular mRNAs. (+) strands destined for encapsidation in new coats are not capped. Three host-encoded anti-viral systems were found, one based on blocking translation of the viral non-polyA mRNAs (Ski2,3,8 complex), another a 5′->3′ exoribonuclease specific for uncapped molecules (such as the viral (+) strands)(Ski1/Xrn1), and the third a mitochondrial nuclease released in cells undergoing meiosis/sporulation (Nuc1). All of these systems protect cells from virus-induced pathology and have clear animal homologs. The 3′ polyA of yeast mRNAs is dispensable for translation in *ski2*Δ *slh1*Δ cells, and such cells are healthy unless the L-A and M dsRNAs are present, suggesting that this polyA is primarily a device allowing cells to distinguish viral and cellular mRNAs. We suggest that the ribosome-associated Ski2,3,8 proteins block 60S subunit joining on polyA^−^ mRNAs. Recent evidence of roles for other cellular components controlling viral expression and replication suggests that yeast viruses will continue to be a fertile area for study of viral pathogenesis and host anti-viral systems.

## 1. Introduction

Alan Bevan discovered that some strains of *S. cerevisiae* secrete a protein toxin that kills other strains, a “killer trait” that showed non-chromosomal inheritance [1,2,3]. A chromosomal gene (*M* or *MMM* and later called *MAK10*) necessary for propagation of the killer determinant was described, as well as “neutral” strains whose non-chromosomal element lacks the killer activity but retains the same immunity to the killer toxin shown by killer strains and requires the same chromosomal gene for its propagation [3].

The killer system has served as a means of studying the processing and secretion of a protein, the toxin, the mechanism of action of the various toxins, and, for us and others, a model to explore the mechanisms of replication of a virus interacting with its “simple” eukaryotic host. Without trying to review the entire field [4], we emphasize several areas of interest beyond the L-A/M virus system studied.

## 2. L-A and M dsRNA Replication (in Brief)

The dsRNAs responsible for the killer phenomenon are a 4.6 kb large dsRNA (L or P1) and a 1.8 kb medium dsRNA (M or P2) [5,6]. There are two L dsRNAs, the more abundant L-A and the less abundant L-BC (L-B and L-C are closely related), stably maintained independent of each other [7]. L-A and L-BC each encode their major coat protein (Gag) and their RNA-dependent RNA polymerase (Pol) [8,9,10]. L-BC has no known role in propagating M dsRNAs. M dsRNAs are “satellites” of L-A, each encoding a protein toxin and an immunity function which is part of the preprotoxin [11]. The viral RNA synthesis (both (+) and (−) strands) takes place in the assembled viral particles [12,13]. L-A (+) strands are extruded from the particles, as are the M (+) strands if the particles are full. M is less than half the size of L-A and a particle can contain two M molecules; a deletion mutant of L-A (X) is less than 1/8 the size of L-A and up to ~8 X dsRNA molecules can occupy a particle [14]. Then, all new X (+) strands are extruded (“head-full replication”). As they leave the particle, (+) strands bound for translation are capped by caps stolen from cellular messages (see “Yeast translation does not need 3′poly(A) of mRNAs”) (reviewed in [4]).

## 3. Violating the Icosahedral Capsid Symmetry Rules

**Figure 1:**
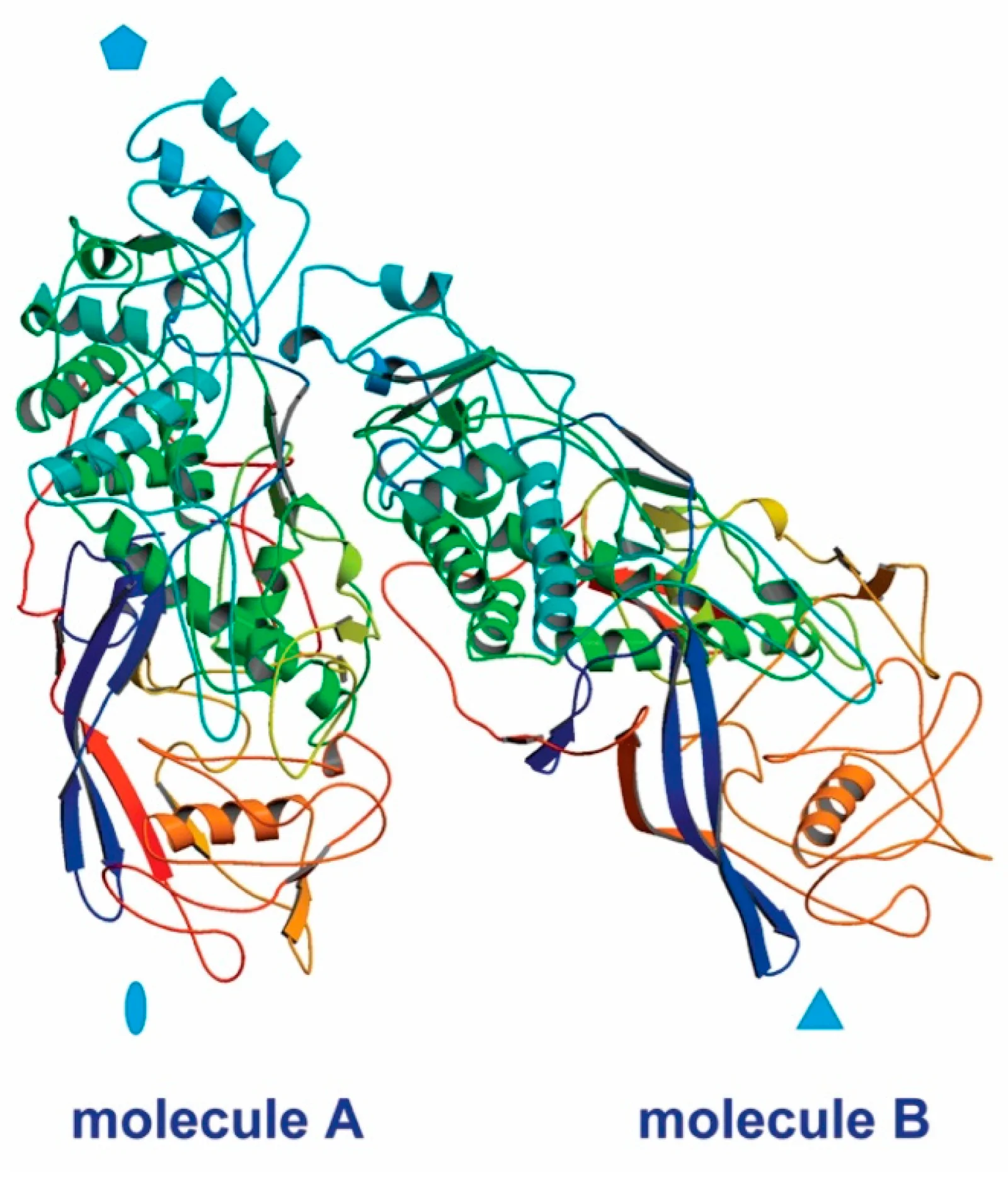
The asymmetric dimer of the L-A virus particle from X-ray crystallographic studies (from [19] with permission). The blue triangle, pentagon and ellipse show the location of the 3-fold, 5-fold and 2-fold axes of the icosahedron relative to the asymmetric dimer of the L-A major coat protein. Type A molecules contact the five-fold and two-fold axes, while type B molecules contact the three-fold axes. The path of the polypeptide chain is colored in a gradient from blue at the N-terminus to red at the C-terminus.

Density and sedimentation properties of the icosahedral L-A viral particles showed that they contained about 120 copies per particle of the major coat protein, Gag [15], a value thought to be in conflict with the symmetry considerations [16,17] which predicted only 60 (T = 1), 180 (T = 3), 240 (T = 4), 420 (T = 7) or higher subunit numbers. We rationalized our result by guessing that there were 60 asymmetric dimers of Gag per particle. CryoEM [18] and X-ray crystallographic [19] studies of the viral particles in the labs of Alastair Steven and Jack Johnson demonstrated that there were two major coat protein forms, one contacting the five-fold and two-fold axes of symmetry, and the other contacting the three-fold axes (Figure 1). In fact, the cores of nearly all dsRNA virus particles have now been found to have this “T = 2” structure [20,21,22,23,24,25]. The exceptions are a *Penicillium crysogenum* virus and the bacteriophage ϕ12, each with a real T = 1 capsid (60 subunits/particle) [26,27].

Why are dsRNA viruses different? While most RNA viruses use the capsid as a simple container to transport and protect the viral genome, dsRNA viruses perform both (+) and (−) strand synthesis within the capsid. In the case of L-A and many other (but not all) dsRNA viruses, the RdRP (RNA-dependent RNA polymerase) is covalently attached to the inner surface of the capsid (as a Gag-Pol fusion protein), and the genome must all pass by this site in order to be transcribed or replicated. These processes require some room for flexibility, and, indeed, it was noted that the density in base pairs/capsid volume of the L-A virus is lower than that known for various double-stranded DNA viruses [28]. The “head-full” limit of packaging is thus not a physical packing limit, but the capsid must be able to release (+) strands before crowding precludes RNA motion past the capsid-bound RdRP.

## 4. Killer vs. RNAi: Is Killer a Benefit or a Detriment?

At first blush, the “killer” toxin secreted by strains carrying M dsRNA would seem to give their hosts a substantial advantage over non-killer yeasts. *S. cerevisiae* lacks an RNAi system, and yeast species found to have dsRNA-based killer viruses all lack RNAi components [29,30]. Expressing RNAi genes from other yeasts in *S. cerevisiae* results in curing of L-A and M1 dsRNAs (but not L-BC (why?)) [30]. It is therefore suggested that *cerevisiae* ancestors (and some other yeasts) lost RNAi in order to have the advantageous killer systems [30]. However, killers are only a few percent of natural isolates, and toxin-resistant strains are similarly rare [31]. If the killer virus were advantageous, most strains would have it, or at least be resistant to the toxin, which is not the case. The toxin is only active at cool temperatures (8–20 °C) and pH near 4.7, and its effects are only evident when the killer strains are at least a large fraction of the cells present. We proposed a different interpretation [32], first proposed for the DNA plasmid-based *Kluyveromyces lactis* killer system, namely that the toxin-immunity functions are a punishment to cells in a colony that have the temerity to lose the plasmid, a sort of hostage situation [33]. A population evolution study of a killer strain showed that in a closed environment, over several hundred generations, point mutations of the M_1_ preprotoxin gene inactivating the toxin but not immunity to the toxin were selected [34]. Further growth selected other point mutations inactivating the immunity function. This showed that making toxin and immunity are each a burden to cells [34]. The low incidence of killer or resistant cells in wild *S. cerevisiae* populations must reflect a balance between the price of making the toxin and immunity proteins and the limited benefit of carrying a wild-type killer virus. It would seem that the limited benefit shown by these results would be insufficient to drive the loss of an RNAi system. Perhaps the killer system only arose and spread in yeasts lacking the RNAi system for other reasons. However, both sides of this argument have some virtue, and further data would be helpful in resolving this issue.

## 5. Anti-Viral Systems in Yeast

Akio Toh-e discovered mutants in four genes of a killer strain that produced a larger killing zone of a sensitive strain than did the parent, calling them “superkillers” (*ski1* (later *xrn1/*…), *ski2*, *ski3*, *ski4*) [35]. Mutants in several more *SKI* genes (*ski6, ski7, ski8*) were found by Porter Ridley and Steve Sommer [36]. *SKI1* was identical to *XRN1* [37] encoding the 5′->3′ exoribonuclease (hence *XRN1*) specific for uncapped single-stranded RNAs discovered by Audrey Stevens and coworkers [38,39]. Deletion of *SKI2, SKI3* or *SKI8* made cells cold-sensitive for growth if the cells carried M dsRNA, but produced no growth defect in the absence of M [36,40,41,42]. This showed that these genes were anti-viral genes, or at least that their most vital function was anti-viral. Ski2p, Ski3p and Ski8p act to prevent translation of non-polyA mRNAs [43,44,45], as discussed further below. The only known non-polyA mRNAs in *S. cerevisiae* are those of the dsRNA viruses L-A, L-BC and M, and the “naked” single-stranded RNA Narnaviruses 20S and 23S RNA, and these (23S RNA has not been examined) are all negatively controlled by these three *SKI* genes [35,46,47]. Overproduction of Ski1p cures the L-A virus [48] and the Narnavirus N1199 [49], again showing its anti-viral nature.

Apparently unrelated to the *SKI* genes, *nuc1* mutants, lacking a mitochondrial nuclease [50], were found to dramatically over produce the L-A virus [51]. Similar overproduction of the L-A virus was found in porin mutants (*por1*), lacking the major mitochondrial outer membrane pore-forming protein [52,53].

**Figure 2:**
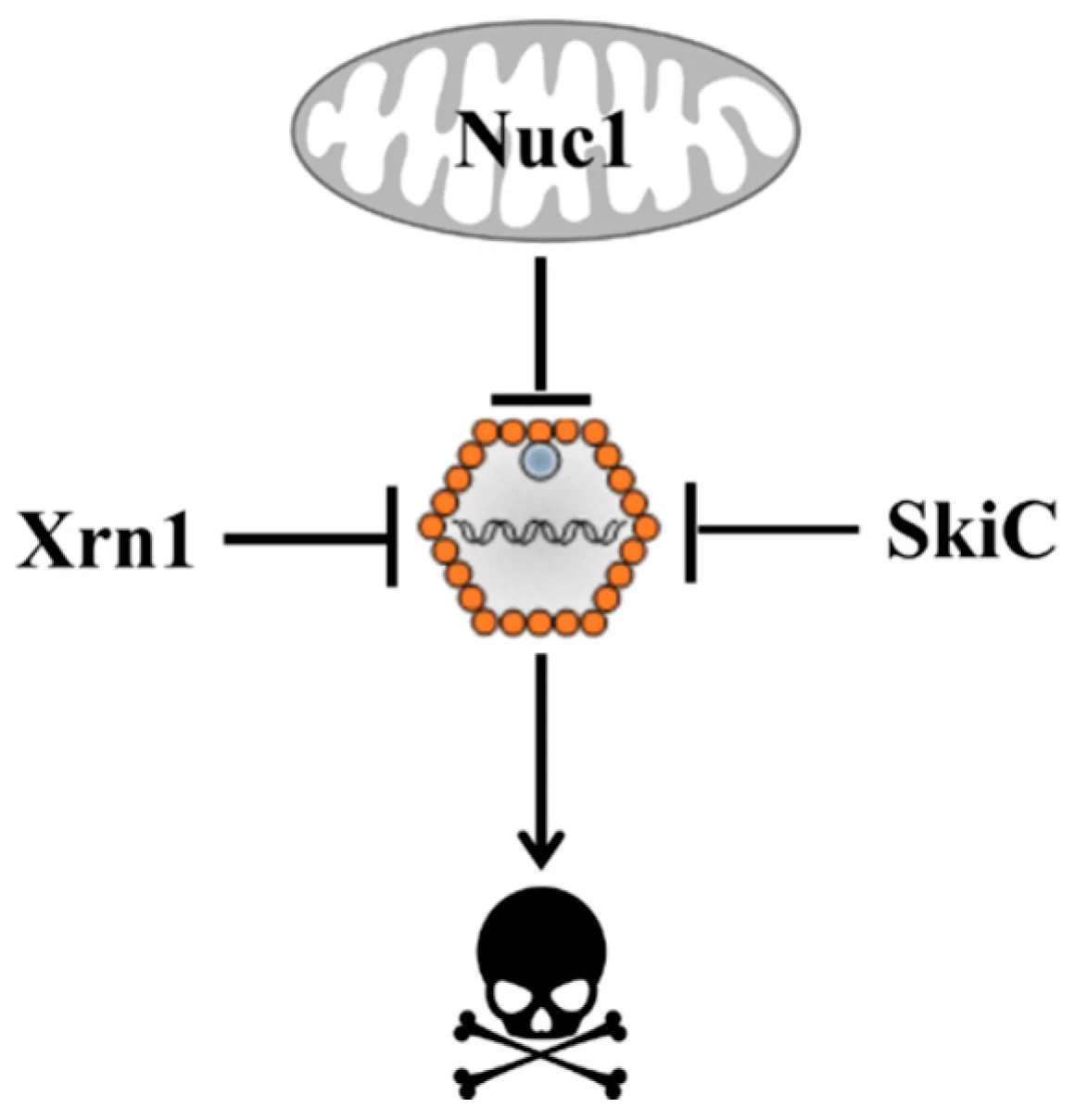
Three anti-viral systems in yeast sufficiently interfere with viral propagation/expression that there is no apparent cytopathology in wild-type infected cells, but multiple mutants expose lethality of the L-A virus and its satellites [56]. SkiC = Ski complex.

Recently, Marc Meneghini’s group has unified many of these results, showing that the porin mutants prevent the mitochondrial Nuc1p nuclease from seeping out of the mitochondria, particularly during meiosis and spore formation [54,55,56]. In the absence of Nuc1p and Ski3p, sporulation is accompanied by massive overproduction of the killer toxin and cell death [54]. Even in the absence of M dsRNA, L-A levels dramatically increase in sporulation of *nuc1*Δ *ski3*Δ diploids, resulting in respiration-deficient spore clones [54]. L-A is also lethal at high growth temperatures in *nuc1*Δ *ski3*Δ cells defective for these two anti-viral systems [56]. There are thus three independent anti-viral systems in yeast (Figure 2). There are mammalian homologs of *SKI2, SKI3* and *SKI8* with similar functions [reviewed in [57]. The Nuc1p nuclease is homologous to EndoG, a mammalian nuclease involved in apoptosis [54], and Gao et al. have suggested that apoptosis originated as an anti-viral process [54].

Chau et al. have expanded the known anti-viral components, showing that *rex2*Δ, *myg1*Δ, or *rnh70*Δ mutants, each defective in a different 3′->5′ exoribonuclease, produce synthetic lethality with *nuc1*Δ *ski3*Δ strains (at normal temperature) that is suppressed by the absence of the L-A virus [56]. Similar results were obtained with components of the SAGA and PAF1 transcription-associated complexes [56]. Overexpression of *SRO9*, *SLF1*, or *PAB1* prevented the growth defect of *nuc1*Δ *ski3*Δ L-A strains at the elevated temperature, and at least *SRO9* and *SLF1* lower the levels of L-A. This shows that these genes have anti-viral activities [56]. Sro9p and Slf1p are homologs of human Larp1 which binds to SARS-CoV-2 RNA and inhibits its replication [58]. Pab1p is the polyA-binding protein.

## 6. Mechanisms of L-A Pathogenesis

The poor growth of cells with elevated levels of L-A [54,56] was correlated with protein aggregation, detected with Hsp104-GFP [56]. This is accompanied by induction of the heat-shock transcription factor, Hsf1, which both protects against the toxic effects of elevated L-A and lowers the levels of L-A in the cells. Does the yeast cell view the L-A particle as an aggregate and try to disaggregate it, thereby distracting chaperones from their usual work? Further work will be needed to understand how elevated L-A levels produce protein aggregation.

## 7. Yeast Translation Does Not Need 3′poly(A) of mRNAs

**Figure 3:**
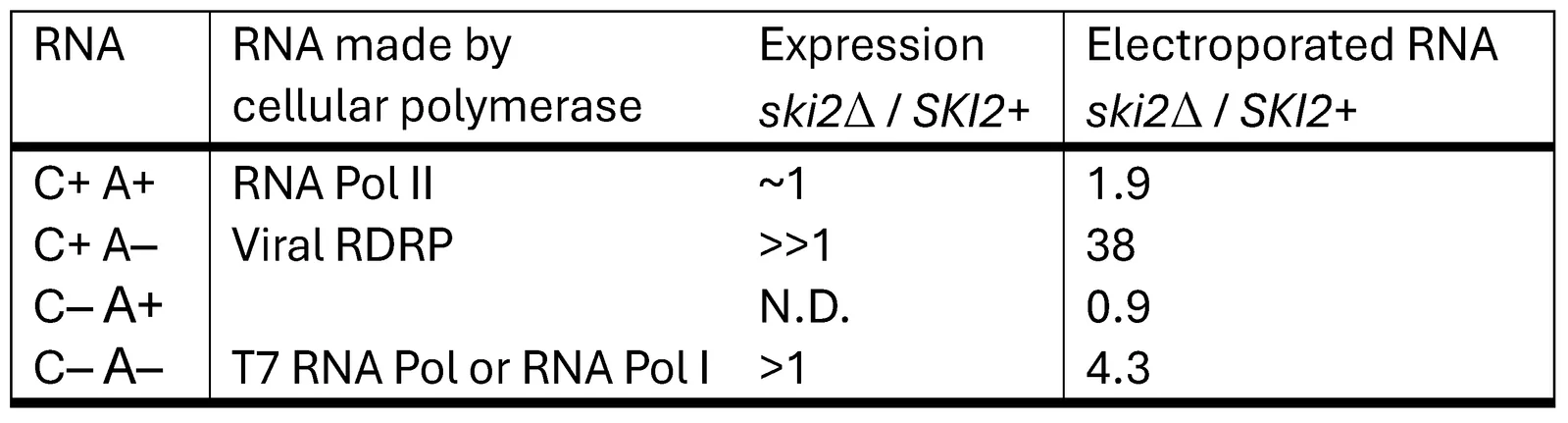
A *ski2* mutation allows translation of non-polyA mRNAs generated in vivo by various RNA polymerases (center column) or in vitro (by T7 RNA polymerase) and electroporated into yeast cells (right column). The presence of 5′ cap (C+) or 3′ polyA (A+) modification of the mRNA is indicated. N.D., not determined. Data from [42,43,44].

Ski2p is an RNA helicase that lowers expression of L-A and M viral transcripts (no polyA) but not expression of the same genes on a DNA plasmid transcribed by cellular RNA polymerase II [42]. Ski2p also acts on genes transcribed from a phage T7 RNA polymerase or from cellular RNA polymerase I (no cap or polyA) [42] (Figure 3, center). These results took on new meaning when Tsutomu Fujimura and coworkers showed that while L-A genomes lack any 5′ cap structure, L-A mRNA is capped by the cap-snatching activity of Gag [59,60,61], an activity discovered in the Sonenberg lab [62,63]. Thus, it is not the absence of cap that is recognized by the Ski proteins (because the L-A or M transcripts actually have a cap), but the 3′ poly(A), missing from L-A genomic RNA and L-A (or M) transcripts, that is critical. Accordingly, when M_1_ dsRNA is supported by the L-A cDNA clone, M_1_ dsRNA copy number shows no effect of a *ski2* mutation because the L-A coat proteins encapsidating and propagating M_1_ are made from a polyA^+^ mRNA made by RNA polymerase II [42]. However, in these cells, a *ski2* mutation results in elevated toxin production (because toxin is made from a viral mRNA lacking 3′ polyA) [42].

**Figure 4:**
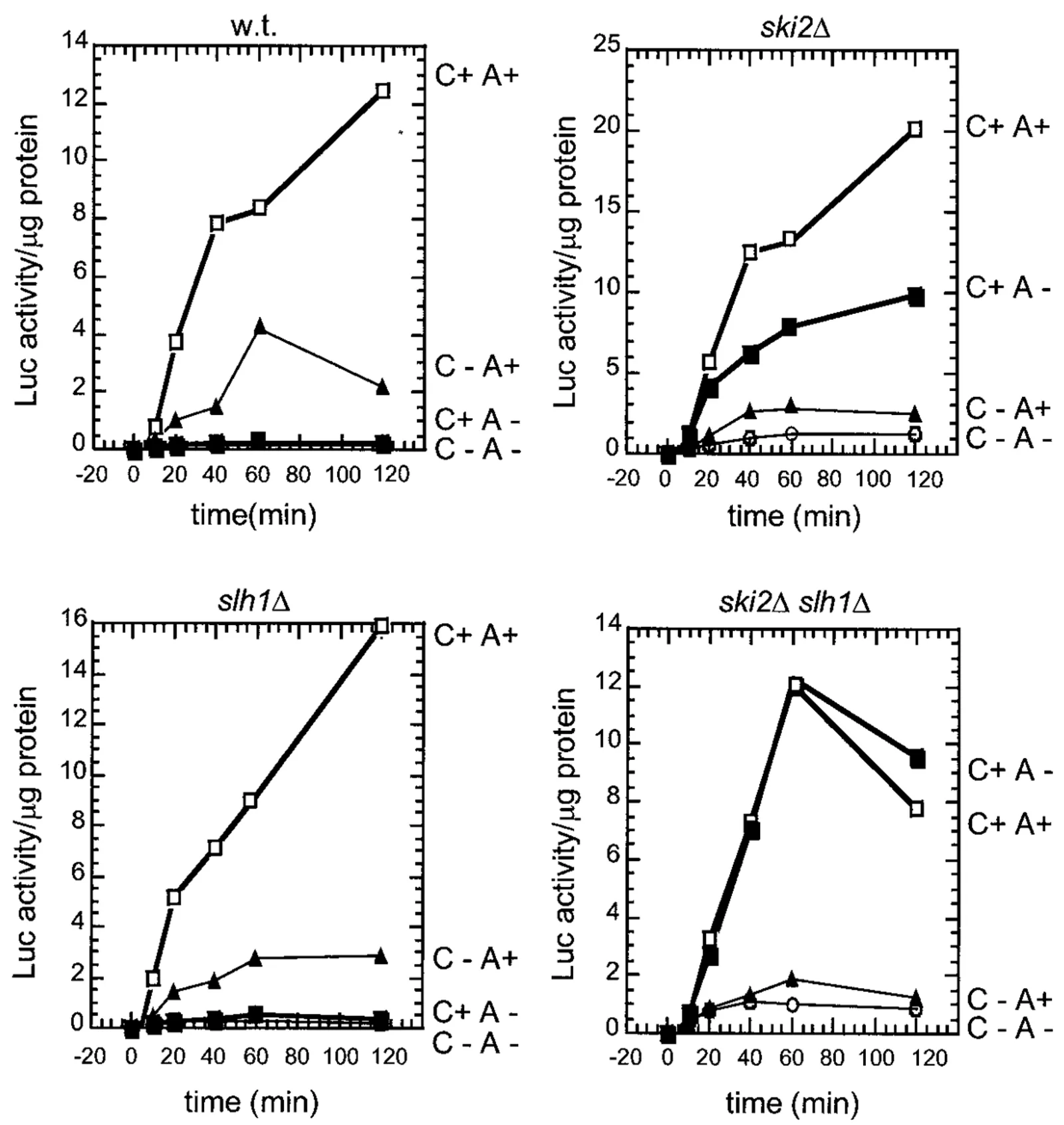
In a *ski2*Δ *slh1*Δ host, non-polyA mRNA is translated with the same kinetics as polyA+ mRNAs (from ref. [44]). Isogenic wild-type, *ski2*Δ, *slh1*Δ, and *ski2*Δ *slh1*Δ strains were electroporated with T7 RNA polymerase-generated RNAs. The results shown are an average of three experiments. (□) C+A+; (■) C+A−; (○) C−A+; and (▲) C−A−.

Direct evidence that the Ski2-3-8 system acts specifically on non-polyA mRNAs came from electroporation of in vitro transcripts of LacZ, with and without either 5′ cap (C) or 3′ polyA (A) or both. Wild-type cells gave ~60-fold better expression of C+A+ mRNA than of C+A− message, while *ski2*Δ cells showed only a ~2-fold preference for C+A+ [43] (Figure 3, right). In contrast, *ski2*Δ did not significantly relieve the strong preference for C+A+ mRNA over C−A+ [43]. A close paralog of *SKI2*, called *SLH1* (Ski2-like helicase), like *SKI2* also affected L-A dsRNA copy number [64]. Anji Searfoss found that *ski2*Δ *slh1*Δ double mutants had massively increased copy numbers of L-A and L-BC dsRNA and did not distinguish electroporated C+A+ from C+A−-mRNAs, producing the same kinetics of protein expression [44] (Figure 4). This showed that, except for the surveillance by the Ski2/Slh1 systems, the yeast translation apparatus does not inherently require 3′ polyA for translation. Similar effects were soon after reported from Arlen Johnson’s lab [45]. Ski2p, Ski3p and Ski8p form a cytoplasmic complex [65] located on the ribosome [66], consistent with an effect on translation.

An in vitro study comparing translation components with and without the human Ski2,3,8 complex showed that the Ski complex released untranslatable model mRNAs from the ribosomes, suggesting that this was the primary action of the ribosome-associated Ski complex [67]. A relation to mRNA 3′ polyA was unfortunately not tested in this study.

An mRNA lacking a stop codon is degraded when the Ski2,3,8 complex releases the stalled mRNA from the ribosome, with Ski7p handing these mRNAs to a group of 3′->5′ exoribonucleases (reviewed by [68]). Presumably non-polyA mRNAs are also released from the ribosome by Ski2,3,8 and share a similar fate. Slh1p is involved in catalysis of the dissociation of 40S and 60S ribosomal subunits from stalled 80S complexes, including ribosomes on an mRNA lacking a stop codon that are affected by Ski2,3,8 [69]. There are no known non-polyA mRNAs in yeast except for those of the dsRNA (L-A, M, L-BC) and ssRNA (20S, 23S and N1199) viruses.

## 8. 60S Ribosomal Subunit Levels Are Critical for M_1_ dsRNA Propagation

M_1_ dsRNA is cured by low concentrations of cycloheximide [70], whose target is ribosomal protein L29. A large series of mutants unable to propagate M_1_ dsRNA (called *mak* for maintenance of killer) were genetically mapped, leading to identification of *MAK8* as the gene encoding large ribosomal subunit protein L3 [71] and *MAK18’s* identity with *RPL41B* encoding L41 [72]. *mak27-1* maps 2 cM (about 6 kb) from *rna1-1* on XIII [73], and *RPL20A* is also 6 kb from *rna1-1*. Examination of ribosomal profiles of these and other *mak* mutants showed that most were relatively deficient in free 60S subunits (compared to 40S), creating half-mer polysomes and reducing translation [74].

We suspected that the absence of 3′ polyA on viral mRNAs might relate to the strict requirement for abundant 60S ribosomal subunits because a then-current view was that the 3′ polyA structure was involved in the 60S joining step of translation initiation [75,76]. Confirming our view, low levels of cycloheximide, that targets ribosomal protein L29, cured M_1_ dsRNA when it is supported by the L-A virus (polyA— mRNA) [70], but not when M_1_ is suppported by the L-A cDNA clone (polyA+) [72]. In fact the L-A cDNA clone suppresses many *mak* mutants now known to affect 60S biogenesis [77]. Mak21p is involved in 60S ribosomal subunit biogenesis, and *mak21-1* mutants show a greater preference for C+A+ over C+A− mRNAs than do wild-type cells [78]. The *mak* mutations producing 60S subunit deficiency are also suppressed for their loss of M dsRNA by *ski2* or *ski3* mutants [79] that derepress translation of polyA— mRNA, again relating Ski2,3,8 to 60S and polyA.

**Figure 5:**
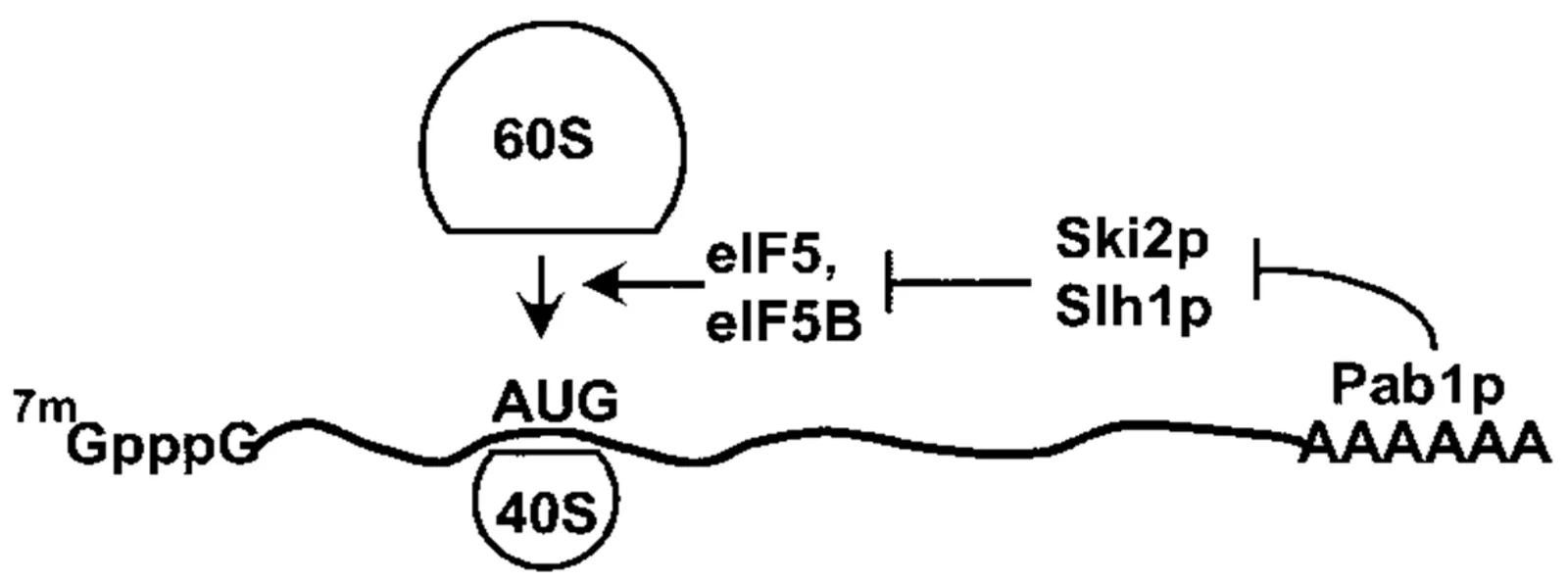
Model of 3′ polyA (and/or Pab1), Ski2-3-8, eIF5B (Fun12p) and 60S subunits in the initiation of translation [83]. The absence of polyA blocks expression, but not in *ski2*Δ *slh1*Δ cells, placing Ski2 and Slh1 downstream of polyA. Expression of a C+A− mRNA is normal in *ski2*Δ *slh1*Δ cells, but not in *ski2*Δ *slh1*Δ *fun12*Δ cells, placing Fun12p/eIF5B (and 60S joining) downstream of Ski2 and Slh1. Lowered 60S subunit levels selectively impair expression of C+A− (e.g., viral) mRNA, but not if the cells are also *ski2*Δ, again suggesting that polyA is involved in 60S joining. mRNAs released from the ribosomes by Ski2-3-8 are degraded.

Initiation factor eIF5B (encoded by FUN12) is critical for the 60S joining step [80,81,82]. A fun12∆ mutation lowers translation of C+A+ mRNA by 93%, but translation of C+A− mRNA is only 20% decreased, suggesting that polyA is important for 60S joining. As a control, a gcd11 mutation (gamma subunit of eIF2) lowers translation of C+A− mRNA as much or more than of C+A+ mRNA. Since the polyA effects on expression are mediated by Ski-Slh, this suggests that Fun12p-catalyzed 60S joining is downstream of Ski-Slh action. The derepression of translation of non-polyA mRNAs in ski2Δ slh1Δ strains is prevented by a fun12Δ mutation, consistent with Ski-Slh preventing translation by blocking 60S joining of non-polyA mRNAs, but is not definitive [83]. The model suggested (Figure 5) has Ski2-Slh1, in the absence of the mRNA’s 3′ polyA, inhibiting eIF5B and eIF5 (perhaps indirectly) in their promotion of the 60S subunit joining the initiation complex [83].

## 9. Kex1, Kex2 and Dissection of Mammalian Hormone Processing

Mutants of a killer strain that lose killer ability include mutants in two chromosomal genes that remained immune to the toxin, but lost the ability to produce killer toxin, and so were named *kex1* and *kex2* (for killer expression [84]). In addition to failing to produce active killer toxin, *kex2* mutants of the α mating type, failed to mate and homozygous *kex2/kex2* diploids failed to go through meiosis and spore formation [85]. The mating defect is, in part, due to a failure of *MAT*α *kex2* strains to produce the α-pheromone [85], a peptide that induces *MATa* cells to arrest in G1 in preparation for cell fusion. Kex1p and Kex2p were then found to encode proteases that cleave at dibasic sequences (e.g., arg-arg) in preprotoxin and prepro-α-pheromone [86,87]. These findings led to the discovery of the homologous mammalian proteases (PC2, PC3 and furin) that process the prohormones of insulin, adrenocortical trophic hormone (ACTH) and other hormones [88].

## 10. Translational Frameshifting in L-A

The sequence of the L-A dsRNA genome [9] revealed two open reading frames, with the 5′ ORF encoding the major coat protein and the 3′ ORF containing consensus sequence patterns described by Kamer and Argos for RNA-dependent RNA polymerases [89]. The 130 bp overlap region contained a possible slippery site [9] similar to that described for Rous sarcoma virus by Jacks and Varmus only a short time before [90]. Jon Dinman showed that this was indeed a slippery site, and that the rules governing frameshifting efficiency were similar to those shown by Jacks & Varmus for RSV [91]. Chromosomal mutants affecting frameshift efficiency (including 5S RNA) or alterations of the “slippery site” with the same effect resulted in loss of M_1_ dsRNA [92,93]. Ribosomal frameshifting proved to be almost as frequent a feature of RNA viruses as of retroviruses, but also appears in some chromosomal genes (reviewed in [94]).

The slippery site, of the form 5′-X XXY YYZ-3′ (gag ORF reading frame shown) is such that tRNAs paired to XXY and YYZ can slip back one base on the mRNA and still have their non-wobble bases properly paired [90]. The RNA pseudoknot just downstream of the slippery site works much better in promoting frameshifting than a simple stem-loop of the same stability [95], a puzzling result at the time. The apparent solution is that while a simple stem-loop will pause the ribosomes in the area of the slippery site, the pseudoknot can precisely pause ribosomes in the right frame at the right place, the “torsional restraint model” [96]. A stem-loop only resists ribosome motion by the stability of a single base pair at a time. However, a pseudoknot presents a barrier stabilized by several base pairs to move beyond the ideal site for frameshifting. Thus, ribosomes can be paused by a pseudoknot in just the right spot for frameshifting much better than by a stem-loop.

## 11. MAK3-MAK10-MAK31, an N-Acetyltransferase Modifying the N-Terminus of L-A Gag Protein

Among the many genes whose mutants lose M_1_ dsRNA, only three lose L-A: *m/mmm/mak10*, *mak3* and *mak31* [3,97,98]. At least Mak3p and Mak10p are not needed for L-BC, the Totivirus closely related to L-A [46]. Mak3p is an N-acetyltransferase modifying the N-terminus of the L-A gag protein, a modification necessary for virus assembly [99,100]. Mak3p recognizes the first four N-terminal amino acid residues of the target protein [101]. Mak3p, Mak10p and Mak31p form a complex with all three subunits required for activity [102,103].

## 12. Natural Variants of L-A and M, and the [HOK]-[NEX] Monster

The biochemistry and molecular genetic studies of L-A and M have brought an understanding of many central mechanisms involved in virus propagation and its control. However, early work with natural variants of L-A, M and chromosomal alleles/genes revealed what may be more subtle interactions whose mechanisms are not yet uncovered. The toxin and immunity functions of various M dsRNAs are reviewed elsewhere [104,105]. These include M_1_, present in many laboratory strains, M_2_ which is found largely in wine yeast strains [106], M_28_ [107] and M_Lus_ [108].

Natural variants of L-A were defined by their interactions with M_1_, M_2_ and chromosomal genes (Table 1). When M_1_ is introduced into an M_2_ strain, M_2_ is selectively lost [109]. L-A-E is a variant of L-A that excludes M_2_ (in the absence of M_1_) [109,110]. L-A-E can propagate M_1_ dsRNA only if the cells have a mutation in *ski2*, one of the anti-virus genes [7,79,110]. Most L-As can propagate M_1_ even in a *SKI+* host, and that ability is called [HOK] (helper of killer; the “H” in “L-A-H”) [7,110]. Some L-As prevent L-A-E from excluding M_2_, an L-A trait called [NEX] (for non-excludable or just N). However, at temperatures of 30 °C or above, [NEX] itself will exclude M_2_ in a cell with a recessive allele of *mkt1* or *mkt2*, two chromosomal genes [111]. Many lab strains have these recessive alleles of *mkt1* or *mkt2. MKT1* encodes a putative nuclease that is ribosome-associated and affects expression of a variety of genes [112,113]. It is not clear how Mkt1p or Mkt2p affect M_2_. Most L-A variants in lab strains have [HOK] and [NEX], but some have only [HOK] or only [EXL]. A single yeast strain had [HOK], [NEX] and, in addition, made several of the 60S-insufficient *mak* mutants able to propagate M_1_, a property called [B] for bypass, and shown to be a property of the L-A in that strain (L-A-HNB) [114].

Just to summarize, there are three ways in which M_2_ can be eliminated. (a) M_1_ will eliminate M_2_, regardless of what L-A is present. (b) L-A-E eliminates M_2_ unless an L-A bearing [NEX] (e.g., L-A-HN) is also present. (c) An L-A with [NEX] (e.g., L-A-HN or L-A-HNB) will eliminate M_2_ in a host bearing a recessive allele of *MKT1* or *MKT2*. The L-A’s found supporting M_1_ can also support M_2_ as long as cells have the dominant alleles of *MKT1* and *MKT2*. Similarly, the L-As in M_2_ strains can support M_1_.

A more detailed molecular view of these interactions would be of interest because tthese are all common natural variants and must reflect natural interactions. However, efforts to launch the L-A virus from a cDNA have not yet succeeded, and expression from cDNA clones is complicated by the presence of 5′ cap and 3′ polyA on clone mRNAs, but no polyA on any viral (+) strands, and no cap on (+) strands destined for encapsidation. As discussed above, these features are central to what we know about viral gene expression and the effects of chromosomal genes.

## 13. [D], a Mysterious Non-Chromosomal Gene, Makes M_1_ More Lethal in ski2 Mutants

Transfer of cytoplasm from a particular K_1_ strain into a *ski2-2* mutant produced only dead or very sick cells [115]. Rosa Esteban’s analysis showed that the ‘disease’ requires a *ski2-2* mutation, M_1_ dsRNA and L-A dsRNA, in addition to the novel cytoplasmic element named [D] for disease [115]. Some strains carry cytoplasmic elements, called [DIN] for D-interference, that prevent the disease, and chromosomal mutants that cannot propagate [D] (called *mad* for maintenance of [D]) were also identified [115]. [D] was shown to depend for its maintenance on L-A, but, unlike [EXL], [NEX] and [HOK], [D] was not located on L-A [115]. [D] was distinct from L-A, L-BC, M_1_, mitochondrial DNA, the [PSI+] prion, 2 micron DNA or 20S RNA [115]. Virus particles from a strain carrying [D] transmit (by an artificial infection method [116]) the [B] (bypass) trait [114], but not [D] [115]. What is [D]? Is the disease it produces related to the other virus-related disease phenomena revealed in *ski* mutants? Might it be related to the Narnavirus N1199 recently discovered in John McCusker’s lab [49]?

## 14. Summary

Studies of the killer virus system have revealed many aspects that proved to be general for the many Totiviruses discovered in recent years, and the broader groups of dsRNA viruses and RNA viruses. Because “Translation is the battle ground of RNA viruses and their hosts”, many host genes and processes affecting the virus have proved to be translation-related: 60S ribosomal subunits, ribosomal frameshifting, Ski2,3,8 affecting translation of viral mRNA, Ski1p that can degrade the uncapped viral RNA as it does uncapped mRNAs, the Mak3-Mak10-Mak31 co-translational N-acetyltransferase and the cap-snatching function of the major coat protein Gag. Future work may be directed at expanding our understanding of the mechanisms at work, and the roles of new anti-viral components protecting the cells. Evidently, the yeast Totiviruses have pathogenic potential, but are controlled by multiple cellular systems. This is likely true of most mycoviruses as well as many mammalian viruses. Many of the anti-viral proteins identified in yeast have human homologs and others have analogs. It may prove possible to manipulate the corresponding human proteins to produce a clinically useful effect.

## Figures and Tables

**Table 1:** Natural variants of L-A and chromosomal genes affecting L-A variants.

L-A Variant	Function	Found in Strain (e.g.,)	References
L-A-E	Excludes M_2_, supports M_1_ only in *ski2*.	AN33	[110]
L-A-HE	Supports M_1_ in *SKI+*, but not M_2_.	200	[111]
L-A-HN	Eliminates M_2_ in *mkt1* or *mkt2* at ≥30 °C; makes M_2_ non-excludable by L-A-E; supports M_1_ in *SKI*+	A364A	[109,111]
L-A-HNB	Suppresses many *mak* mutant effects Superkiller phenotype in *SKI+* host; has properties of L-A-HN	P28-24C	[79,114]
**Non-chromosomal gene**			
[D] = disease	Exacerbates the disease produced in *ski2* mutants by M dsRNA.	P28-24C	[115]
**Chromosomal genes**			
*mkt1*, *mkt2*	Dominant alleles, *MKT+*, needed for maintenance of M_2_ (K_2_) in strains with [NEX]	AN33, 18	[109,111]
*makx*	Maintenance of killer: N-acetyltransferase OR 60S subunit biogenesis.		See text
*skix*	Superkiller. Ski proteins block expression of non-polyA mRNAs.		[35]

In the designations of L-A subtypes, E = [EXL] (excluder of M_2_), N = [NEX] (no exclusion by [EXL]), H = [HOK] (helper of killer) and B = [B] (bypass of 60S deficiency effect) (see text).

## Data Availability

No new data were created or analyzed in this study. Data sharing is not applicable to this article.
